# Multiple *p-n* junction subwavelength gratings for transmission-mode electro-optic modulators

**DOI:** 10.1038/srep46508

**Published:** 2017-04-18

**Authors:** Ki Young Lee, Jae Woong Yoon, Seok Ho Song, Robert Magnusson

**Affiliations:** 1 Department of Physics, Hanyang University, Seoul 133-791, Korea; 2 Department of Electrical Engineering, University of Texas – Arlington, Arlington, TX 76019, United States

## Abstract

We propose a free-space electro-optic transmission modulator based on multiple *p-n*-junction semiconductor subwavelength gratings. The proposed device operates with a high-Q guided-mode resonance undergoing electro-optic resonance shift due to direct electrical control. Using rigorous electrical and optical modeling methods, we theoretically demonstrate a modulation depth of 84%, on-state efficiency 85%, and on-off extinction ratio of 19 dB at 1,550 nm wavelength under electrical control signals within a favorably low bias voltage range from −4 V to +1 V. This functionality operates in the transmission mode and sustainable in the high-speed operation regime up to a 10-GHz-scale modulation bandwidth in principle. The theoretical performance prediction is remarkably advantageous over plasmonic tunable metasurfaces in the power-efficiency and absolute modulation-depth aspects. Therefore, further experimental study is of great interest for creating practical-level metasurface components in various application areas.

Leaky-mode resonances in nanopatterned thin-film structures are of interest owing to their great potential for creating integration-compatible, multifunctional devices harnessing desired spectral, polarization, intensity, and phase properties^1^,^2^,^3^. Guided-mode-resonance elements^1^, high-contrast gratings^2^, and plasmonic metasurfaces^3^ have been extensively studied within this context. Adding active tunability to these device classes for applications in practice, various approaches have been suggested using thermo-optic effects^4^, micro-electro-mechanical system architectures^5^, and liquid-crystal-based index-tuning methods^6^. Further expanding the application areas and innovating classical device counterparts, a major line of research is presently pursuing higher tuning speed, smaller device footprint area, and better long-term stability in order to secure essential requirements in potential application areas including ultra-broadband optical signal processing, high-power laser machining, and compact LIDAR systems.

To this end, free-carrier-induced electro-optic (EO) effects in heavily doped semiconductors and transparent conducting oxides at epsilon-near-zero (ENZ) conditions have been extensively studied as an efficient tuning mechanism. In particular, ENZ nanofilms incorporated in metal-oxide-semiconductor (MOS) capacitor arrays have showed remarkable intensity and phase modulation properties driven by field-effect free-carrier accumulation and depletion^7^,^8^,^9^,^10^. In this approach, highly dissipative, deep-subwavelength plasmonic resonances are necessary to induce significant optical interaction with sub-10-nm-thick EO-active layers. Consequently, strong absorption and low resonance Q factor result in performance restrictions such as shallow signal modulation depth, low absolute efficiency, and poor high-power durability. In addition, it is presently unclear whether or not the plasmonic MOS capacitor approach using highly reflective metallic components as an indispensable constituent material can operate in the transmission mode which is desirable for variety of applications.

Pursuing high-performance tunable leaky-mode resonance devices operating in the transmission mode in this paper, we propose an approach based on high-Q guided-mode resonances (GMRs) in low-loss semiconductor nanogratings. The proposed device class consists of moderately doped, low-loss semiconductor *p-n* junctions in a resonant subwavelength grating structure as shown in Fig. 1(a–d) where basic operation scheme, device structure, and electrical connections, and a possible architecture for integrated modulator arrays are illustrated, respectively. This structure is designed such that a high-Q GMR is supported in the optical domain while in the electrical domain bias voltage across the multiple *p-n* junctions effectively control density of mobile electrons and holes, resulting in the associated tuning of the Drude-type optical dielectric constant^11^. The major advantage of this configuration over the plasmonic tunable metasurfaces is to sustain a low-loss, high-Q resonance feature that experiences a remarkable resonance center shift under a favorable bias-voltage region. Hence, desired properties such as high efficiency and high on/off extinction ratio can be supported in the transmission mode operation. While the proposed operation principle is applicable for various group IV and III-V-compound semiconductor materials, here we select an interleaved Si *p-n* junction nanograting architecture as one promising example. Using well-established electrical and optical modeling methods, we theoretically demonstrate a robust transmission modulator with on/off power ratio of 18.9 dB, on-state efficiency of 85.2%, and modulation bandwidth of 54.3 GHz at an operation wavelength of 1550 nm. These performance characteristics are driven by favorably small bias voltage values in a range of −4 V ~ +1 V and possibly maintained in the high-speed operation regime up to 50 GHz when appropriate in-plane miniaturization schemes are incorporated.

## Results

### Free-carrier-induced EO effect in multiple *p-n* junction layers

**Figure 1:**
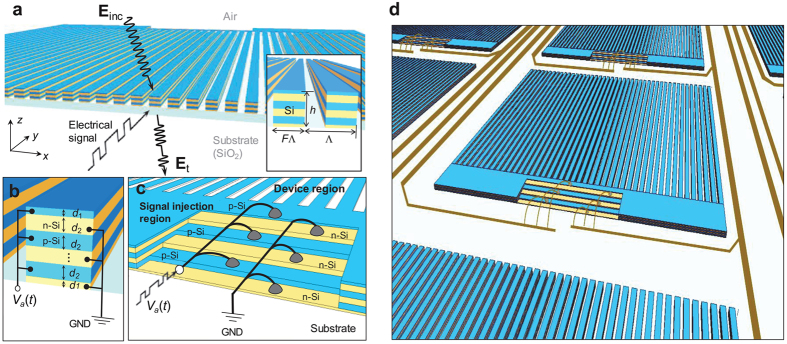
Schematic of a Si *p-n* junction subwavelength-grating intensity modulator. (**a**) Device operation concept and parameters. (**b**) Multiple *p-n* junction configuration embedded in the grating structure. *V*_*a*_ denotes applied bias voltage for inducing the free-carrier-induced EO effect. (**c**) Bias-voltage injection scheme for simultaneously driving constituent Si lines in an isolated device region. (**d**) Illustration of the proposed EO modulator arrays integrated on a single substrate surface.

The EO effect of our interest in this paper is based on change in mobile electron and hole densities that we denote by *N*_*e*_ and *N*_h_, respectively, in response to an applied bias voltage *V*_*a*_ across the *p-n* junctions. Dielectric constant *ε*_Si_ of Si in the optical domain is determined by a Drude formula


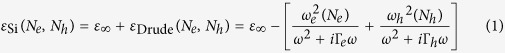


where *ω*_*e*,*h*_ and Γ_*e*,*h*_ denote plasma and collision frequencies, respectively, for electron (*e*) and hole (*h*) plasmas, and *ε*_∞_ = 11.7. Following the canonical Drude model, *ω*_*e*,*h*_^2^(*N*_*e*,*h*_) = *N*_*e*,*h*_*e*^2^(*ε*_0_m^*^_e,*h*_)^−1^ with the elementary electric charge *e*, vacuum permittivity *ε*_0_, and effective mass m^*^_e,*h*_. In moderately doped Si with donor and acceptor doping concentration of *N*_0_ = 10^18^ cm^−3^ (=*N*_*e*_ = *N*_*h*_), for example, values for these parameters are m^*^_e_ = 0.27*m*_*e*_, m^*^_h_ = 0.39*m*_*e*_, *ω*_*e*_ = 0.449 eV/*ħ*, Γ_*e*_ = 4.95 × 10^−2^ eV/*ħ, ω*_*h*_ = 0.374 eV/*ħ*, and Γ_*h*_ = 5.36 × 10^−2^ eV/*ħ*^12^. The two frequency-dependent parts in the Drude-part dielectric constant *ε*_Drude_ describe optical response of the mobile electron and hole plasmas, respectively. Under applied bias voltage *V*_*a*_ across the *p-n* junctions in the configuration in Fig. 1(b,c), mobile electrons and holes are redistributed to modify *N*_*e*_ and *N*_*h*_, and hence the *ω*_*e*_ and *ω*_*h*_ values until the internal electric field energy becomes minimal. Therefore, the desired bias-voltage-dependent dielectric constant is obtained in this interaction process. Previously, a similar effect provides a robust tuning mechanism for high-speed, low power consuming in-line Si-photonic modulators with device footprint length scales in the order of 1 mm to 100 μm^13^,^14^. In our case, the effect is used to create an electrically tunable low-loss GMR element taking advantage of the resonant light confinement in a subwavelength-thick zero-order grating layer.

Rigorously treating this free-carrier-induced EO effect, the carrier-density *N*_*e*_ and *N*_*h*_ distributions in thermal equilibrium follow the Poisson-Boltzmann distribution^15^. We use a 2-dimensional finite-element-method model^16^ of the Poisson-Boltzmann equation to calculate bias-voltage-dependent *N*_*e*_(*y, z*) and *N*_*h*_(*y, z*). We assume *d*_1_ = 55 nm, *d*_2_ = 2*d*_1_ = 110 nm, and identical donor (*N*_*n*_) and acceptor (*N*_*p*_) concentrations such that *N*_*p*_ = *N*_*n*_ = *N*_0_ = 10^18^cm^−3^ in this calculation. In this multiple-junction structure, we include five pairs of alternating 110-nm-thick *p-n* junction cells in the order of *np-pn-np-pn-np* from bottom. The thickness of 110 nm for a single *p-n* junction unit cell is chosen such that the depletion layer at a bias voltage *V*_*a*_ = −4 V well below the breakdown voltage of −5.54 V for the given doping concentration completely covers the entire device volume and consequently the mobile-carrier-induced EO effect occurs over the entire device. The peculiar feature of *d*_2_ = 2*d*_1_ is a natural consequence of this layer-design rule to maximize the EO effect with a favorably minimal material embodiment in the proposed device concept.

**Figure 2:**
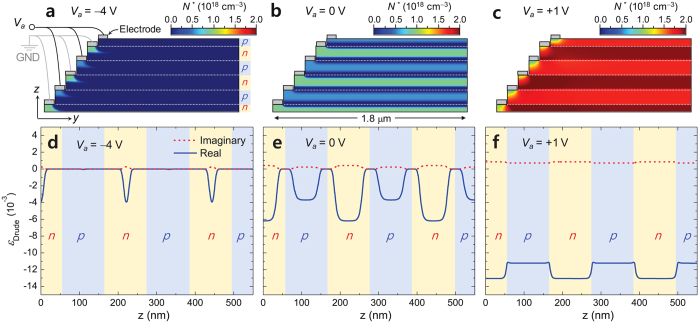
Free-carrier induced electro-optic effect in the proposed multiple *p-n* junction structure. Effective compound-carrier density N^*^(*y, z*) distribution at room temperature (300 K) for *V*_*a*_ = (**a**) −4 V, (**b**) 0 V, and (**c**) +1 V. Electrode connection for bias-voltage application and *p*/*n* layers are indicated in (a). We assume *d*_1_ = 55 nm and *d*_2_ = 110 nm. Drude-part dielectric constant *ε*_Drude_(*z*) profiles for *V*_*a*_ = (**d**) −4 V, (**e**) 0 V, and (**f**) +1 V. Blue solid and red dashed curves correspond to Re(*ε*_Drude_) and Im(*ε*_Drude_) profiles, respectively, and the *p*/*n* layers are indicated on the background of each panel.

Results for three bias-voltage values of *V*_*a*_ = −4 V (reverse bias), 0 V (neutral), and +1 V (forward bias) are shown in Fig. 2(a–c), respectively. Therein, we indicate distribution of an effective compound-carrier density N^*^ = (m^*^_e_m^*^_h_)^−1/2^(m^*^_h_*N*_*e*_ + m^*^_e_*N*_*h*_) that we define as an intuitive, single density parameter directly relevant to the free-carrier EO effect. This definition is followed by a simpler expression for *ε*_Drude_ as


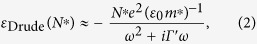


where m^*^ = (m^*^_e_m^*^_h_)^1/2^ and Γ′ = (m^*^_h_*N*_*e*_Γ_*e*_ + m^*^_e_*N*_*h*_Γ_*h*_)(m^*^_h_*N*_*e*_ + m^*^_e_*N*_*h*_)^−1^ in the weak collision regime where Γ_*e*,*h*_ ≪ *ω* and Γ_*e*,*h*_ ≪ *ω*_*e*,*h*_. Obviously, *ε*_Drude_ is linearly proportional to N^*^. The *V*_*a*_-dependent N^*^ distributions in Fig. 2(a–c) show that N^*^ is effectively modified from 0 to 2 × 10^18^cm^−3^ over the whole structure under *V*_*a*_ adjustment in a range from −4 V to 1 V. In addition, N^*^ is almost independent of in-plane position *y*, confirming that the electrical signal-injection configuration envisioned in Fig. 1(c) should be feasible for efficiently controlling N^*^ over the whole isolated device region. Subsequent change in *ε*_Drude_ at a wavelength of 1,550 nm is in a range from 0 to −1.3 × 10^−2^ for Re(*ε*_Drude_) and from 0 to 0.86 × 10^−4^ for Im(*ε*_Drude_) as shown in Fig. 2(d–f). Therefore, the proposed configuration provides a 10^−2^-order EO change in Re(*ε*_Drude_) over a 550-nm-thick Si film under a favorably low bias-voltage tuning range from −4 V to +1 V while keeping acceptably low material absorption levels of Im(*ε*_Si_) < 10^−3^.

### EO-tunable GMR excitation

**Figure 3:**
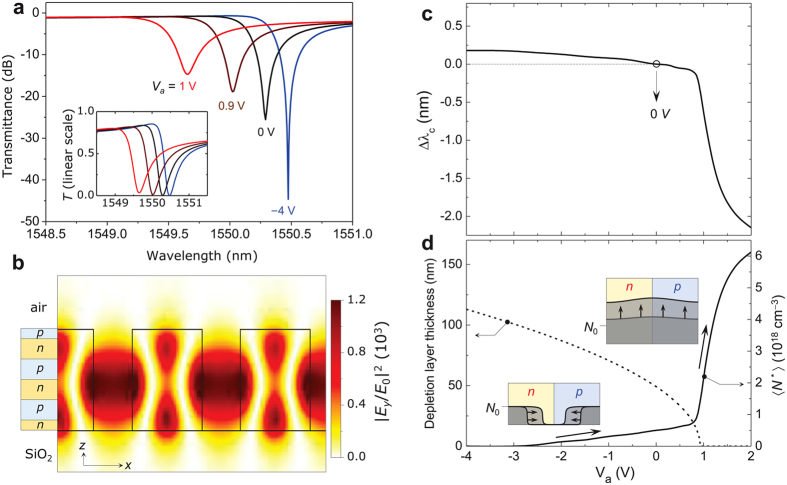
EO-tunable high-Q GMR excitation. (**a**) Transmission spectra of an example device for *V*_*a*_ = −4.0 V (blue), 0 V (black), 0.9 V (brown), and 1.0 V (red). The optimized device parameters are Λ = 730 nm, *F* = 0.647, *h* = 550 nm. Normal incidence (*θ* = 0) of TE-polarized planewave is assumed. The *p-n* junction configuration is identical to that used in Fig. 2. Inset shows the transmission spectra in linear scale. (**b**) Electric field intensity |*E*_*y*_|^2^ distribution at the resonance center (λ_0_ = 1550.02 nm) for *V*_*a*_ = 0 V. (**c**) Resonance-center shift Δλ_c_ as a function of applied bias voltage *V*_*a*_. (**d**) Average effective compound carrier density 〈N^*^〉 (solid curve, right-vertical axis) and depletion layer thickness (dashed curve, left-vertical axis) and in response to the applied bias voltage control. Two inset diagrams illustrate carrier distribution statics in the open (lower left) and closed (higher right) depletion-layer regimes.

We apply the obtained bias-voltage-induced mobile-carrier effect to an example GMR element optimized for the transmission-mode optical modulation in the telecommunications C-band around 1,550 nm. Figure 3(a) shows the *V*_*a*_–dependent transmission spectra in dB (a linear scale in the inset) under transverse-electric (TE) polarized planewave incidence at surface-normal angle (*θ* = 0). The optimized grating-design parameter values are given in the caption. Following the standard convention, the TE polarization refers to electric field oscillating in the axis of grating lines (*y*-axis). We use the finite-element method^16^ in this calculation involving *ε*_Si_(*z*) profiles obtained by the method described in the previous section. The transmission spectra show an asymmetric Fano-resonance profile as a result of the configuration interference between resonant and non-resonant pathways^17^. The resonant pathway is created by coupling of the incident wave with a leaky TE_0_ mode and its radiation decay toward the transmitted zero-order planewave channel through dominant first-order diffraction processes. This resonance feature possesses remarkably high resonance Q factor ~3.69 × 10^3^. Consequently, the design yields a very high field enhancement factor ~1.2 × 10^3^ in the 550-nm-thick EO-active Si-*p-n*-junction layers as confirmed in Fig. 3(b) showing an electric-field intensity distribution at the resonance center wavelength.

Subtle interaction between the highly enhanced resonant optical fields and bias-voltage-induced mobile-carrier effect results in a resonance-center (λ_c_) shift Δλ_c_ as shown in Fig. 3(a). Basically, the observed resonance shift is directly resulting from the *V*_*a*_-dependent change in *ε*_Si_. Although there is no exact closed-form expression for the dielectric-constant-dependent resonance shift known in general, a plausible estimation can be found by taking the Wentzel-Kramers-Brillouin (WKB) approximation on the resonance condition. In our case, the change in *ε*_Si_ leads to the change in the optical path length inside the Si grating bars while there is no optical-path-length difference outside. For a small dielectric-constant change, i.e., Δ*ε*_Si_ ≪ 1, that further implies no significant modification in the field distribution of the leaky resonance mode, the WKB approximation for the eigenvalue determination^18^ dictates that the optical phase accumulation inside the Si bars should remain constant under small change in *ε*_Si_. This condition directly yields a relation (*n*_Si_ + Δ*n*_Si_)^−1^(λ_c_ + Δλ_c_) = *n*_Si_^−1^λ_c_, where *n*_Si_ = *ε*_Si_^1/2^ and consequently Δ*n*_Si_ = (2*n*_Si_)^−1^Re(Δ*ε*_Si_) = (2*n*_Si_)^−1^ Re(Δ*ε*_Drude_) as the dielectric constant change is solely in the Drude part. Including Eq. (2) and the standard phase-matching condition Λ^−1^ = *n*_eff_λ_c_^−1^ for a GMR at normal incidence, where *n*_eff_ is effective index of the leaky guided mode, the constant optical-phase-accumulation condition is rewritten by


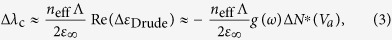


where *g*(*ω*) = *e*^2^[*ε*_0_m^*^(*ω*^2^ + Γ′^2^)]^−1^. Obviously, increase in N^*^ (ΔN^*^ > 0) with *V*_*a*_ leads to a corresponding linear blue shift of the resonance feature or vice versa.

From Fig. 3(a), we find that the resonance-center shift in response to the applied bias voltage *V*_*a*_ has two different regimes. In Fig. 3(c), we show Δλ_c_(*V*_*a*_) that reveals slow blue shift with increasing *V*_*a*_ in the low-voltage region of *V*_*a*_ < 1 V and abrupt increase in the differential resonance shift Δλ_c_/Δ*V*_*a*_ in the high-voltage region of *V*_*a*_ > 1 V. According to Eq. (3), this peculiar property is associated with the dependence of N^*^ on *V*_*a*_. *V*_*a*_–dependent volume average 〈N^*^(*V*_*a*_)〉 of the effective compound-carrier density is plotted in Fig. 3(d) and it is in exact correlation with −Δλ_c_(*V*_*a*_) in Fig. 3(c). Explaining the dependence of 〈N^*^〉 on *V*_*a*_ in Fig. 3(d), a key factor is built-in potential *V*_built-in_ across the *p-n* junction. Applying the Poisson-Boltzmann equation for *V*_*a*_ = 0 in our case with *N*_*p*_ = *N*_*n*_ = *N*_0_ = 10^18^ cm^−3^, we obtain *V*_built-in_ = 0.934 V. In the low bias-voltage region of *V*_*a*_ < *V*_built-in_, increase in 〈N^*^〉 with *V*_*a*_ is led by the decrease in the depletion layer thickness without significant growth in the carrier-density level. In contrast, in the high bias-voltage region of *V*_*a*_ > *V*_built-in_, the depletion region is closed and the excessive electrons and holes injected from the electrodes lead to mobile-carrier density level growth in the whole Si regions to result in a more rapid increase in 〈N^*^〉 with *V*_*a*_. In Fig. 3(d), we provide the *V*_*a*_-dependent depletion layer thickness and illustrations showing the two regimes of carrier distribution statics.

**Figure 4:**
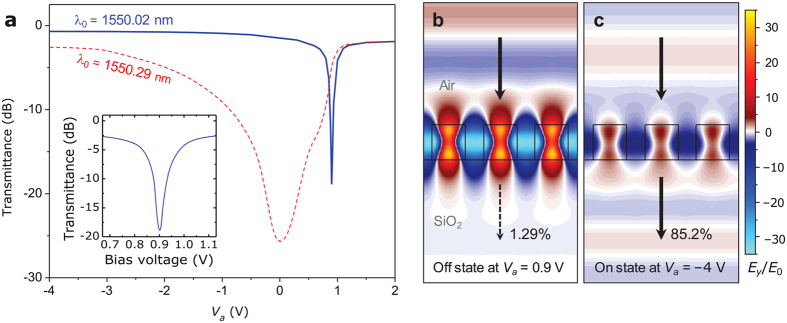
Transmission modulation properties. (**a**) Bias-voltage-dependent transmittance at operation wavelength of λ_0_ = 1550.02 nm (blue solid curve) and 1550.29 nm (red dashed curve) for an optimized device used in Fig. 3. Inset shows magnified plot for λ_0_ = 1550.02 nm. Total electric field *E*_*y*_/*E*_0_ patterns for the (**b**) off-state and (**c**) on-state operation regimes at *V*_*a*_ = 0.9 V and −4 V, respectively. We assume λ_0_ = 1550.02 nm for (**b,c**).

Importantly, the obtained resonance-shift tunability in Fig. 3(c) implies a full λ_c_ tuning range over 2.3 nm that is remarkably larger than the resonance bandwidth of 0.43 nm by a factor 5.3. Therefore, we can fully utilize the spectral maximum and minimum as the on-state and off-state transmittance levels, respectively. For our particular design at an optimal wavelength of λ_0_ = 1550.02 nm which corresponds to the transmittance minimum at *V*_*a*_ ≈ *V*_built-in_, sharp transmission modulation is obtained as shown in Fig. 4(a). In this case, the intensity modulation is induced mainly in the closed depletion-layer regime and thereby small bias-voltage tuning induces rapid intensity change as confirmed again in the total electric field patterns for *V*_*a*_ = 0.9 V and −4.0 V in Fig. 4(b,c). Key performance parameters in this case are transmittance modulation depth of 83.9%, on-state efficiency of 85.2%, and on-off extinction ratio of 18.9 dB under remarkably low control bias-voltage signals within the −4 ~ +0.9 V range. Importantly, the obtained performance parameters are highly desirable for variety of applications when compared with LiNbO_3_-crystal-based EO modulators requiring 100 V-scale control signals for similar performances.

For a different operation wavelength which corresponds to the transmittance minimum at *V*_*a*_ < *V*_built-in_, we have much slower intensity tuning as the device operates in the open depletion-layer regime. For example, we select λ_0_ = 1550.29 nm and the corresponding *V*_*a*_-dependent transmittance is indicated by red dashed curve in Fig. 4(a). Such slow intensity modulation is desirable for continuous modulation or precise control of light intensity for analog signal processing systems while the rapid, closed depletion-layer regime is more appropriate for digital signal processing applications.

**Figure 5:**
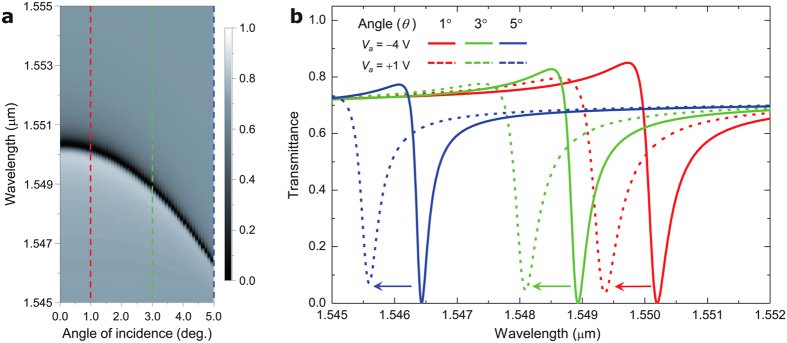
Angle dependence of the EO-tunable GMR. (**a**) Angle-dependent transmission spectrum for a fully depleted case at *V*_*a*_ = −4 V. (**b**) Bias-voltage-dependent spectral profiles of the transmittance at angles *θ* = 1° (red), 3° (green), and 5° (blue). Solid and dashed curves indicate the spectral profiles for *V*_*a*_ = −4 V and +1 V, respectively.

The proposed device is basically a 1D-periodic GMR element and thereby has a characteristic angular dispersion. In our case, a primary angular dispersion of the resonance location appears for the angle of incidence with respect to *x* axis on which the discrete light diffraction processes take place. As the resonance location follows the dispersion curve of the leaky guided mode, the angular shift of the resonance wavelength as a function of polar angle *θ* of incidence can be found from the definition of group velocity, i.e., *V*_*x*_ = ∂*ω*(**k**)/∂*k*_*x*_, where *V*_*x*_, *ω*(**k**), and *k*_*x*_ denote group velocity in *x* axis, dispersion frequency surface, and *x*-component wavevector of the leaky guided mode, respectively. A simple calculus with basic relations of *ω*(**k**) = 2π*c*λ_c_^−1^, *k*_*x*_ = 2π*c*λ_c_^−1^sin*θ*, and the diffractive phase matching condition λ_c_^−1^sin*θ* = *n*_eff_λ_c_^−1^−Λ^−1^ results in


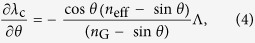


where *n*_G_ = *c*/*V*_*x*_ is group index of the leaky guided mode. For near-normal incidence (*θ *≪ 1), Eq. (4) reduces to ∂λ_c_/∂*θ* ≈ − *n*_G_^−1^*n*_eff_Λ. This property explicitly appears in the angle-dependent transmission spectrum as shown in Fig. 5(a) where the angle dependent λ_c_ loci are identified as being along the dark transmission dip. Therein, ∂λ_c_/∂*θ* = 0 at *θ* = 0 as the group index *n*_G_ diverges to infinity for the laterally standing guided-mode with *V*_*x*_ = 0 at normal incidence. For off-normal incidence (*θ* ≠ 0) for which the two counter-propagating guided modes are not coincidental anymore and the resonance is driven by a single leaky-guided mode. Consequently, the group-to-effective-index ratio *n*_G_^−1^*n*_eff_ tends to a constant value and so is ∂λ_c_/∂*θ*. Estimating from Fig. 5(a), the off-normal angle-tunability ∂λ_c_/∂*θ* ≈ −2 nm/deg. at *θ* = 5°. Importantly, the angle-dependent shift of λ_c_ does not significantly affect the resonance profile and the EO-tunable resonance shift as shown in Fig. 5(b). In particular, the EO tunability of 0.17 nm/V persists for all three cases and the transmission modulation depth values are 85%, 83%, and 77% for *θ* = 1°, 3°, and 5°, respectively.

The angular dispersion of λ_c_ and persistent EO tuning properties suggest important information for operation of the proposed device class in practice. First, highly collimated light beam should be used to fully utilize the proposed device functionality. Angular full-width-at-half-maximum bandwidth for the GMR in our case is estimated from Fig. 5(a) as 2.6° at *θ* = 0 and 0.18° at *θ* = 5°. Divergence angle of the incident light beam should be well within these values. In another consideration, the angle tunability of λ_c_ provides an efficient way to precisely shoot the desired operation wavelength. In practice, fabrication errors and imperfections present. Although exact tolerance values depend on fabrication steps and specific tools selected for device production, one may accept a-few-nm scale errors in the spatial device parameters and grating period and a-few-Å scale errors in the layer thickness values. A combination of these errors in period, grating linewidth, and layer thicknesses might result in an imperfect λ_c_ off from the desired value by an amount even in a 10-nm scale. Considering 0.1 nm (distributed feedback type) ~10 nm (Fabry-Pérot type) for typical diode laser line width around 1,550 nm, the anticipated fabrication errors can be critically problematic if there is no tuning method available for matching λ_c_ to the source-laser wavelength. Suppose that we control *θ* within a ±10° range and with an accuracy of 10^−3^ deg. The angle tunability of 2 nm/deg. implies a full tuning range of ~40 nm with an accuracy of ~2 pm. We note that typical angle precision of commercially available rotary or tilting stages for optomechanical controls is in 10^−4^ deg. scales.

Further considering applicability of the proposed device concept in practice, modulation bandwidth is an important measure. The GMR bandwidth and RC time constant are two major factors in this consideration. For the analyzed example device in Figs 3 and 4, the estimated GMR bandwidth is Δ*f*_opt_ ~ 54.3 GHz. Therefore, stable 10-GHz-scale optical signals can be generated in a purely optical property aspect. However, the final modulation bandwidth should be also restricted by electrical response characteristics, i.e., a bias-voltage signal modulation bandwidth Δ*f*_bias_ = *τ*_RC_^−1^, where *τ*_RC_ denotes the RC time constant determined by the junction capacitance and termination impedance. Assuming the standard radio-frequency termination impedance of 50 Ω and the design configuration used in Figs 2 and 4, estimated Δ*f*_bias_ values are 14.7 MHz for a device footprint area of 1 × 1 mm^2^. Since Δ*f*_bias_ is inversely proportional to device footprint area, GHz-scale modulation bandwidth should be feasible for small devices with the footprint area <260 × 260 μm^2^. We note that Inoue *et al*.^19^ recently demonstrated a high-Q GMR filter with a device footprint area reduced down to 10 × 10 μm^2^ without significant degradation in the spectral performance characteristics by using graded-parametric design approach combined with integrated first-order Bragg reflection boundaries. Introducing such miniaturization strategy to the proposed concept, Δ*f*_bias_ > Δ*f*_opt_ and the full resonance bandwidth should be available for the final optical signal-modulation bandwidth.

## Discussion

In summary, we proposed a multiple-*p-n*-junction subwavelength grating structure that enables high-performance EO modulation in the transmission mode. The proposed device operates under high-Q GMRs interacting with electric signals through the Drude-type optical free-carrier effect. Using rigorous electrical and optical modeling methods, we theoretically demonstrated highly efficient transmission modulation generated by remarkably low-voltage control signals with modulation speed in possibly 10 GHz scales. Notably, the obtained properties are supported by the low-loss free-carrier-induced EO effect occurring in the whole device region with 500-nm-thick Si layers as opposed to the transparent-conducting-oxide-based plasmonic metasurface approaches involving a sub-10-nm-thick EO-active region and strong ohmic absorption. In another similar approach, a low-loss GMR modulator was suggested using a combination of the Burstein-Moss effect, Pockels effect, and Fraz-Keldysh effect in a weakly-modulated InGaAsP waveguide grating structure^20^,^21^. Therein, a robust reflection modulation with an extinction ratio of 17 dB and modulation bandwidth of 5 MHz was experimentally demonstrated.

Experimental realization of the proposed device concept is definitely the next step. In potential fabrication, crucial parts are to establish 100-nm-thick multiple *p-n* junction cells and subwavelength grating structure with a critical dimension in a few 100 nm scale. First, the multiple *p-n* junction cells can be generated by the standard deposition processes based on chemical vapor deposition and sputtering techniques. We note that the present state-of-the-art deposition methods easily produce such multiple-junction semiconductor layer structures as established well in tandem solar cells and in vertical-cavity surface-emitting lasers^22^,^23^,^24^. Second, the subwavelength periodic structure for a high-Q resonance excitation is also well-established using standard nanolithography techniques including the laser-interference lithography^4^ and electron-beam lithography^24^.

Considering further study, applicability and limitations of the proposed concept over other spectral domains are of key importance. Although direct application of the concept to the visible domain is unclear because of highly lossy nature of the group IV or III-V-compound semiconductors, longer-wavelength applications in the mid-infrared (mid-IR) and THz domains are intriguing in several aspects. First, we notice from Eq. (2) that the EO modulation of *ε*_Drude_ scales with λ^2^. This implies that the modulation amplitude Δ*ε*_Drude_ that is in the order of 10^−2^ around λ = 1.5 μm under Δ*V*_*a*_ = 5 V is amplified up to the unity order around λ = 15 μm in the mid-IR domain and even further up to the order of 10^2^ around λ = 150 μm in the THz domain. Therefore, the proposed device class can take advantage of the far stronger EO effect in the mid-IR and THz domains. In addition, fabrication errors and imperfections with respect to the operation wavelength are substantially lower and consequently precise device fabrication is much more feasible in the longer wavelength domains. Therefore, further in-depth study on the available materials, parametric optimization, and experimental realization in the telecommunications IR and longer wavelength domains is of great interest to develop compact, low driving power, and high-speed modulators for applications in telecommunications, optical information processing, LIDARs, laser machining, and many others.

## Additional Information

**How to cite this article:** Lee, K. Y. *et al*. Multiple *p-n* junction subwavelength gratings for transmission-mode electro-optic modulators. *Sci. Rep.* **7**, 46508; doi: 10.1038/srep46508 (2017).

**Publisher's note:** Springer Nature remains neutral with regard to jurisdictional claims in published maps and institutional affiliations.
